# Roles and impact of pharmacy technicians on hospital wards: a systematic review

**DOI:** 10.1016/j.ijnsa.2026.100593

**Published:** 2026-06-10

**Authors:** Marjan De Graef, Jolien Heirman, Tamara Snoeck, Eibert R. Heerdink, Nienke E. Dijkstra, Tinne Dilles, Brecht Serraes

**Affiliations:** a Clinical Nursing & Allied Health Research and Development Group (CNuAH-RD), Nursing and Paramedical Department, Vitaz Hospital and Health Care, Moerlandstraat 1, 9100 Sint-Niklaas, Belgium; b Nurse and Pharmaceutical Care (NuPhaC), Universiteitsplein 1, 2610 Antwerp, Belgium; c Department of Nursing Science and Midwifery, Centre for Research and Innovation in Care (CRIC); Faculty of Medicine and Health Sciences, University of Antwerp, Universiteitsplein 1, 2610 Antwerp, Belgium; d Research Group Sustainable Pharmaceutical Care, University of Applied Sciences Utrecht, PO Box 12011, 3501 AA Utrecht, the Netherlands; e Division of Pharmacoepidemiology and Clinical Pharmacology, Utrecht Institute for Pharmaceutical Sciences, Utrecht University, PO Box 80082, 3508TB Utrecht, the Netherlands; f University Centre for Nursing and Midwifery, Department of Public Health and Primary Care, Faculty of Medicine and Health Sciences, Ghent University, Corneel Heymanslaan 10, 9000 Ghent, Belgium

**Keywords:** Pharmacy technicians, Medication safety, Nursing workload, Interprofessional collaboration, Ward-based care, Quality of care, Healthcare workforce

## Abstract

**Background:**

Medication management on hospital wards is increasingly supported by pharmacy technicians, yet their specific tasks and effects have not been comprehensively synthesised in the literature.

**Objective:**

To synthesise international evidence on the tasks performed by pharmacy technicians on hospital wards and to evaluate the effects on patient-, pharmaceutical-, nursing-, and ward-related outcomes.

**Information sources:**

Five databases (EMBASE, CINAHL, Web of Science, PubMed, ScienceDirect) were systematically searched to November 2024, supplemented with hand searches.

**Methods:**

This systematic review included studies that reported quantitative outcomes of pharmacy technicians performing medication-related tasks on general wards. Screening, data extraction, and quality appraisal (CASP) were performed independently, and findings were synthesised narratively due to clinical and methodological heterogeneity.

**Results:**

Twenty-one studies met the inclusion criteria. Pharmacy technicians most frequently performed medication history review and reconciliation (11 studies), followed by support in preparation and administration (5 studies), supply and dispensing management (4 studies), and discharge education (3 studies). Across these domains, their involvement was consistently associated with improved patient safety and workflow outcomes. Thirteen studies reported reductions in medication errors, with technicians identifying and correcting omissions, dosing errors, and prescribing discrepancies. Eight studies demonstrated gains in pharmacy staff efficiency through more accurate medication histories and optimised pharmacist time, while six studies highlighted improvements in nursing efficiency, including shorter medication rounds and fewer interruptions. Staff satisfaction increased in five studies, and cost-effectiveness analyses (3 studies) indicated neutral or positive financial impact through reduced missed doses and lower wastage. Patient satisfaction, reported in one study, improved through discharge education, with patients expressing greater understanding of their medications.

**Conclusion(s):**

This systematic review identified a broad range of tasks performed by pharmacy technicians on hospital wards. Although several studies reported positive effects on patient-, pharmaceutical-, nursing-, and ward-related outcomes, the overall methodological quality was moderate and substantial heterogeneity existed in pharmacy technician roles, tasks and outcomes measured. Overall, the evidence suggests consistent beneficial effects across multiple outcome domains, although the strength of inference is limited by methodological heterogeneity and study design limitations. Further high-quality, standardised research is needed to determine the magnitude and generalisability of these effects.

**What is already known**

• Ward-based medication processes are time intensive and error prone.
• Pharmacy technicians increasingly support medication related tasks.

Alt-text: Unlabelled box dummy alt text

**What this paper adds**

• Pharmacy technicians improve medication safety and efficiency in hospital wards.
• Training gaps and weak study designs limit generalisable conclusions.
• Benefits are consistent across multiple outcome domains.

Alt-text: Unlabelled box dummy alt text

## 1 Introduction

Healthcare systems worldwide continue to face increasing service demands, workforce shortages, and growing complexity in patient care (World Health Organization, 2022). These pressures intensify the need for workforce optimisation strategies that ensure safe and efficient medication management within hospital settings (Shaffer et al., 2022). Pharmacy technicians have therefore attracted increasing attention as a potential workforce component that may support both pharmacists and nursing staff, particularly on hospital wards where medication processes are highly resource-intensive (Kjeldsen et al., 2023; Summerlin et al., 2024). A time–motion observational studies have shown that nurses spend approximately 25% of their working time on medication administration and a similar proportion on communication-related activities, underscoring the central role of these processes in daily ward practice (Kellett et al., 2024; Keohane et al., 2008). These findings emphasise the importance of optimising medication-related workflows in increasing the workforce on hospital wards.

The International Pharmaceutical Federation (FIP), representing a global network of pharmacy professionals, has highlighted the importance of strengthening and effectively deploying the pharmacy support workforce within its workforce Development Goals (International Pharmaceutical Federation, 2022). These goals emphasise the value of structured roles, competency development, and the integration of technical support staff to enhance medication management systems. Such policy frameworks demonstrate international interest in expanding pharmacy technician roles, yet they do not provide empirical evidence on how pharmacy technicians are currently utilised on hospital wards or what the measurable effects of their involvement are.

Medication management on hospital wards is frequently hindered by inefficiencies, interruptions, and errors. Nurses spend a substantial proportion of their time preparing, dispensing, administering, and documenting medications – activities associated with workflow fragmentation, increased cognitive load, and elevated risk of medication-related incidents (Johnson et al., 2017; Westbrook et al., 2011). In response, several countries have explored the integration of pharmacy technicians into medication-related ward processes to improve logistical efficiency, enhance accuracy, reduce interruptions, and alleviate nursing workload. Various studies have described pharmacy technician involvement in these processes, yet it remains unclear which specific tasks pharmacy technicians perform on hospital wards and which outcomes are most relevant for evaluating their contribution. Recent qualitative research from Belgium reflects growing interest in expanding pharmacy technician roles and provides insight into nurses’ and pharmacy technicians’ experiences with such models (De Graef et al., 2024b). While these contextual findings are informative, they highlight the broader need for a systematic international overview of ward-based pharmacy technician tasks and their associated effects on patient, pharmaceutical, nursing, and ward-level outcomes.

Despite increasing attention to pharmacy technician deployment on hospital wards, the evidence base required to support informed workforce planning remains underdeveloped. It is not yet clearly established which medication-related tasks pharmacy technicians undertake across different clinical settings, nor is there a consolidated understanding of the outcomes used to assess their contribution or the effects associated with their involvement. This lack of clarity limits the ability of healthcare organisations, educators, and policymakers to design or implement evidence-informed task redistribution models that strengthen medication management and support nursing and pharmacy teams.

To address these gaps, the present systematic review aims to synthesise international evidence on the tasks performed by pharmacy technicians on hospital wards and to evaluate the effects on patient-, pharmaceutical-, nursing-, and ward-related outcomes. By providing a structured and evidence-based overview, this review seeks to inform workforce optimisation strategies, support the safe and effective integration of pharmacy technicians into ward-based medication management, and identify priorities for future empirical research.

## 2 Methods

### 2.1 Study design

This systematic review aimed to synthesize evidence on the role of pharmacy technicians in medication-related tasks on nursing wards in general hospitals. A review protocol was registered in the PROSPERO database (CRD42023465217) prior to the conduct of the review, ensuring transparency and methodological rigor (De Graef et al., 2024). The reporting of this review followed the PRISMA guidelines (Page et al., 2021).

### 2.2 Search strategy

Five electronic databases were consulted up to November 2024: EMBASE, CINAHL– EBSCOhost interface, Web of Science, PubMed - MEDLINE, and ScienceDirect. Search strategies combined key terms and their variations: pharmacy technician, pharmacy assistant, pharmacy support, or dispensary technician, combined with hospital, nursing ward, hospital ward, or nursing ward. The full search strategies, including complete search strings for each database, database-specific syntax, and all applied limits are provided in Supplementary Table S1.

No restrictions were applied regarding publication date. However, only full-text journal articles published in English, French, or Dutch were considered for inclusion. Language and full-text availability were applied as search filters in the database searches. In addition to database searches, reference lists of included studies and relevant reviews were screened, and conference records identified through database searches were considered to identify additional relevant studies. Grey literature (such as theses, institutional evaluations or unpublished reports) was not systematically searched, as the review focused on peer-reviewed quantitative studies to ensure methodological consistency and comparability of outcomes. Trial registries and professional organisation websites were not systematically searched.

To enhance completeness, an additional quality assurance step in the search process was implemented. Screening was supplemented using AS Review (a machine learning–assisted screening tool), combined with manual reference list checks to reduce the likelihood of missing relevant studies retrieved through database searches (Verdonschot et al., 2025).

### 2.3 Eligibility criteria

Nursing wards in general hospital settings were eligible. These were defined as adult or paediatric inpatient units providing ward-based hospital care, including internal medicine, general surgery, orthopaedics, geriatrics, rehabilitation, general paediatrics, and both adult and paediatric intensive care units. Settings that do not reflect ward-based inpatient care—such as emergency departments, operating theatres, maternity services and military hospital units—were excluded because they differ substantially in workflow organisation, interprofessional collaboration and medication system structures.

Medication-related tasks were defined a priori as any ward-based activities directly contributing to the safe, accurate and efficient management of inpatient medications. This broad definition encompassed all forms of medication-related work that pharmacy technicians may perform on inpatient wards, without specifying task types in advance. Eligible tasks were required to relate directly to inpatient medication management and to be evaluated using quantitative outcomes.

### 2.4 Study selection

Decisions regarding the inclusion of studies were reached by consensus among the authors. Studies were included if they reported primary quantitative results on pharmacy technicians performing medication-related tasks within nursing wards in general hospital settings. Eligibility was assessed using the predefined inclusion and exclusion criteria described above.

After removal of duplicates, titles and abstracts were independently screened for eligibility by three authors (MDG, JH, BS). The full texts of potentially relevant studies were then assessed independently by the same three authors to confirm inclusion. In cases where the three reviewers did not reach agreement during the full-text screening, the study was discussed within the project group (MDG, TD, BS, EH, ND) to arrive at a final decision. Studies were considered eligible if they examined tasks performed by pharmacy technicians, including studies that compared these tasks with those traditionally carried out by other healthcare professionals; pre-post designs without a comparator were also eligible to capture implementation effects in real-world settings.

### 2.5 Data extraction and synthesis

Data were extracted independently by two reviewers (MDG, TS) using a standardised extraction form based on the Cochrane data extraction template, which was modified to align with the specific objectives of this review (Sambunjak et al., 2017). Extracted data included study characteristics (authors, year, country, design), descriptions of the roles and tasks of pharmacy technicians on hospital wards, contextual information, participant characteristics, intervention details and reported outcomes.

Following independent extraction, the two reviewers compared their results. Discrepancies between the extracted data were resolved through discussion until consensus was reached.

A narrative synthesis was conducted to summarize and interpret the findings. Studies were grouped and compared based on the types of tasks performed by pharmacy technicians and the nature of the reported outcomes. Due to the heterogeneity of reported outcomes and the diversity of practice settings among the included studies, a meta-analysis was not performed. Extracted data were organised into summary tables presenting study characteristics, intervention details, and reported outcomes.

To structure the narrative synthesis, medication-related tasks were subsequently grouped into two broad categories: (1) *logistical medication tasks*, referring to activities that support the physical preparation, supply, distribution or organisation of medicines at ward level (e.g., stock management, supply checks, ward based dispensing), and (2) *clinical or patient facing tasks*, referring to activities involving direct patient interaction or clinical decision support (e.g., medication history taking, medication reconciliation, discharge related communication). These examples are intended to illustrate the distinction and were not used as eligibility criteria. The two-category structure served only as an organising framework to organise the synthesis and comparability across studies.

### 2.6 Quality assessment

The methodological quality of the included studies was independently assessed by two reviewers (MDG, TS) using the Critical Appraisal Skills Programme (CASP) checklist for quantitative studies (CASP Checklist);Singh, 2013). A brief calibration exercise was conducted on two studies prior to the full assessment to ensure consistency between reviewers. Studies were rated as having low, moderate, or high methodological quality based on predefined criteria. Any disagreements between reviewers were resolved through discussion or, when necessary, by consultation with a third reviewer (BS).

To ensure consistency in quality appraisal, a structured scoring framework was developed based on these CASP domains. This approach aligns with recommendations from Long et al. (2020), who reviewed the practical application of the CASP tool and highlighted opportunities to improve its clarity and usefulness in evidence synthesis. Their insights support the domain-based classification used in this review and reinforce the importance of consistent interpretation across studies and study designs.

Overall study quality was classified according to the following criteria:

• High quality: At least five domains rated ‘✓’, or four domains rated ‘✓’ and two ‘?’, provided no critical methodological flaw was present (i.e., no ‘✗’ in the methodology domain).
• Moderate quality: Three to four domains rated ‘✓’, or higher ratings accompanied by a ‘✗’ in the methodology domain.
• Low quality: Two or fewer domains rated ‘✓’, or the presence of serious flaws across multiple domains.

Critical flaws were defined as methodological limitations that substantially compromised a study’s internal validity. These included, for example, the absence of a necessary comparator group, major selection bias (e.g., convenience sampling with unclear representativeness), insufficient reporting of essential methodological procedures (such as how medication discrepancies were verified), or serious concerns regarding the reliability of outcome measurement. Studies with such flaws were not categorised as high quality, irrespective of the total number of CASP domains rated positively (Long et al., 2020).

This framework enabled the differentiation of studies with robust methodological foundations from those with limitations or uncertainty, while ensuring consistency across varying study designs. The quality assessment results were considered in interpreting the findings; however, study quality was not used as a criterion for exclusion in order to retain a comprehensive overview of the available evidence while transparently accounting for methodological limitations.

## 3 Results

### 3.1 Study selection

A total of 5085 records were identified from the search of five databases. After removing duplicates, the 3878 records were screened for relevance based on titles and abstracts. Most of these records were excluded at this stage, primarily because they did not meet the inclusion criteria regarding study setting, population, or design. Many studies described tasks performed by pharmacy technicians within the hospital pharmacy rather than on hospital wards, or were conducted in non-hospital settings such as primary care.

**Fig. 1:**
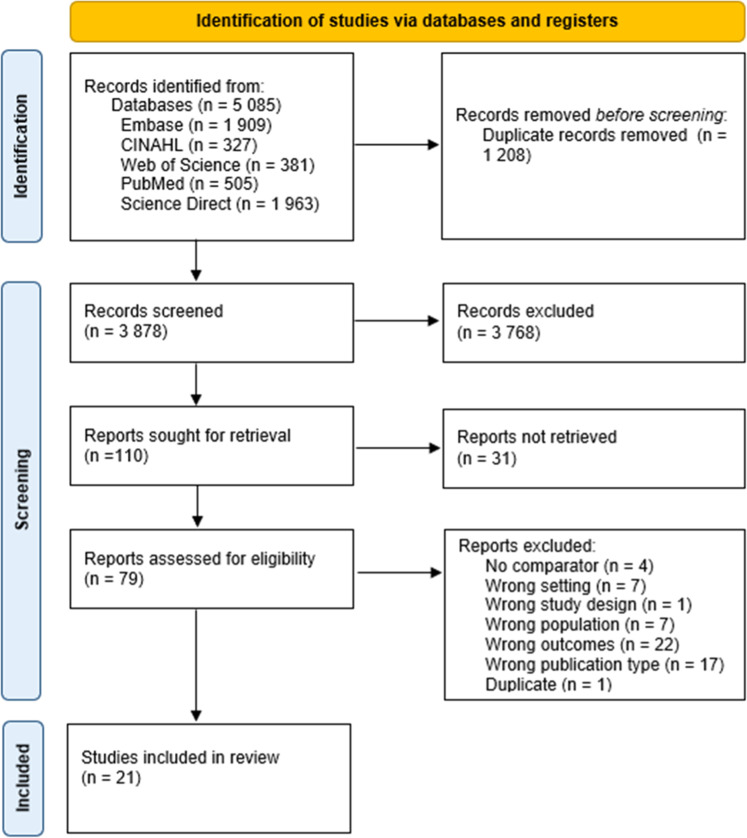
PRISMA 2020 flow diagram of the screening process.

Of the remaining 110 full-text records were assessed for eligibility, 21 studies met the inclusion criteria and were included in this systematic review. The manual hand search of reference lists did not yield any additional studies, while AS Review identified one further eligible study, which is included in the total of 21 studies (Wagner et al., 2010). The included studies were published between 2010 and 2023. The full selection process is detailed in the PRISMA flow diagram (Fig. 1).

### 3.2 Study quality

The methodological quality of the included studies was appraised using the Critical Appraisal Skills Programme (CASP) checklists for cohort studies (n = 19) and randomized controlled trials (RCTs, n = 2). The majority of cohort studies fulfilled most CASP criteria; however, several exhibited limitations, particularly regarding control of confounding and completeness of follow-up. For the two RCTs, all core CASP quality domains were adequately addressed.

Table 1 presents a detailed summary of the quality appraisal for each study. Study quality was evaluated across six key domains: (1) clarity of the research question, (2) methodological appropriateness, (3) control of confounding, (4) adequacy of follow-up, (5) reliability of results, and (6) applicability of results to the local context. Each domain was rated as ‘✓’ (criterion met), ‘?’ (unclear or partially met), or ‘✗’ (not met).

**Table 1:** Summary of the quality appraisal per study.

### 3.3 Main findings

The terminology used to describe the role of the pharmacy technician varied considerably across studies, including titles such as pharmacy technician, pharmacy assistant, ward-based pharmacy technician, pharmaconomist, advanced pharmacy technician, and medicine assistant (defined as pharmacy assistants working on inpatient wards). The studies originated from the United States, the United Kingdom, Denmark, Australia, and Canada, reflecting international variation in both role designation and scope of practice. Additionally, the educational level and training requirements for pharmacy technicians differed substantially across countries – details reported in Table 2 where available – further influencing the breadth and complexity of delegated tasks observed. For consistency, the term pharmacy technician will be used throughout this paper to refer to all of these roles.

**Table 2:** Details of included studies – pharmacy technicians’ tasks.

Authors, Year (country)	Setting	PTs tasks	Description PT task	Frequency PTs tasks observed	Comparator healthcare professional	Pre-service training of PTs	Additional training PT before intervention
Anderson et al., 2021)(Aus)	Elective short-stay surgery centre in a tertiary-referral teaching hospital.	Patient discharge education	Medication Education Technician (MedTech) providing medication education at discharge.	100 patients MedTech educated	Pharmacist	Certificate III and IV in Hospital/Health Services Pharmacy support.	Training and competency module: 50 h of training and assessment over 13 mornings
(Baqir et al., 2015) (UK)	2 wards (surgical & medical) at a general hospital.	Medication preparation & administration support	Pharmacy assistants (PA) – supported medicine administration: double-checking, sourcing medication, identifying medication boxes, and reminding nurses to sign inpatient treatment charts.	2 weeks during the 8 am and 1 pm medicine administration rounds. (181 patients and 1 427 doses due)	Nurse	Not reported	Onward training: importance of not missing medication, prompting nurses when a medication is missed, ensuring medication is available
(Buck et al., 2013) (Denmark)	Department of geriatric medicine, University Hospital.	Medication history review & reconciliation	PTs conducted medication reconciliation on the time of patients’ hospital admission & daily medication reviews on all patients.	7 week period: PT present Mo–Fri between 8 am and 3 pm (212 reconciliations and 936 prescribing reviews)	Pharmacist/ Physician & Nurse	Not reported	Not reported
(Chan et al., 2015) (Canada)	Cardiology ward at a paediatric tertiary care hospital.	Medication history review & reconciliation	Medication reconciliation. On admission (verifying medication history), on transfer (reconciliation of transfer medication orders)	3 week period: PT available Mo–Fri between 8 am and 4 pm (27 medication histories, 20 admission reconciliation, 34 transfer reconciliation)	Pharmacists and/or nurse	Not reported	Pt trained for 5 days by the pharmacy resident and clinical pharmacist to conduct medication history and medication reconciliation.
(El-Fahimi et al., 2020) (UK)	An emergency assessment unit of a large district hospital.	Medication preparation & administration support	PTs role involved: administration of drugs, pharmacy returns, ensuring that medicines were moved with patients, second-checking, highlighting drugs that were not available to the pharmacy team.	30-day period (PT was present from 6 am to 22 pm)	Nurse	Not reported	Not reported
(Elliott et al., 2014) (Aus)	A subacute aged care service at a major public hospital.	Medication history review & reconciliation	Ward pharmacy technician (WPT) medication reconciliation on admission, screening medication charts and calculating creatinine clearance values for the pharmacists, and preparing information for patient education.	13-week period (195 patients)	Pharmacist	The WPT had completed a ‘pharmaceutical assistant’ course in Malaysia	Not reported
(Hwang et al., 2021) (Aus)	A small regional hospital.	Medication history review & reconciliation	Ward-based pharmacy technicians (WBPT) conducted medication histories on admission, performing adverse drug reaction and allergy audits, and patient bedside drawers audits.	5 months period (1524 patients for medication history interview) & 1 month period (medication reconciliation)	Pharmacist	Certificate IV in Hospital/Health Services Pharmacy Support	14-day training program (medication history-taking technique, structuring the patient interview process, understanding the workflow process) & ongoing assessment every 3 months.
Authors, Year (country)	Setting	PTs tasks	Description PT task	Frequency PTs tasks observed	Comparator healthcare professional	Pre-service training of PTs	Additional training PT before intervention
(Jobin et al., 2018) (USA)	A non-profit regional hospital.	Medication history review & reconciliation	PTs gathered a medication history during the inpatient admission process.	2-months period (101 patients)	Pharmacist	PTs are licensed and must successfully pass a national certification exam within the first year of employment.	10-day training; competency is assessed by a supervising PT.
(Kjeldsen et al., 2023) (Denmark)	Geriatric ward at a general hospital.	Medication supply & dispensing management	All tablets and capsules were dispensed by the pharmaconomists (=PTs) after the ward round at noon to cover the time period of 6 pm to 12 am the next day.	2 weeks	Nurse	Not reported	Not reported
(Kram et al., 2019) (USA)	Intensive care units in a large academic medical centre.	Medication history review & reconciliation	Medication histories completed by PTs	3 weeks (176 medication histories)	Pharmacist	Not reported	Not reported
(Kramer et al., 2014) (USA)	An acute care hospital.	Medication history review & reconciliation	Admission medication histories are taken by certified pharmacy technicians.	2 months (153 medication histories)	Pharmacist/ nurse	Not reported	Not reported
(Lloyd, 2020) (UK)	Medical wards in a large acute hospital.	Patient discharge education	PTs transcribed medications from an inpatient paper-based chart into an electronic discharge system including documentation of allergy status, and if an item was new, stopped or amended during admission.	5-day period (Mo – Fri)	Physician	Band 5 Agenda for Change pharmacy technicians (accredited medication accuracy checkers)	1-hour workshop as training in transcribing of medications (selection of correct drug, dose, frequency and duration)
(Nguyen et al., 2017) (USA)	University affiliated, non-profit hospital	Medication history review & reconciliation	Obtaining admission medication histories by pharmacists-supervised pharmacy technicians (PSPTs) for patients at high-risk drug events.	30 obtained medication histories (12 by pharmacists and 18 by PSPTs)	Pharmacist	Not reported	Didactic training (8–16 h): admission medication history workflow, patient interview, documentation & Experiential training: 5 observations and 5 proctors
(Raleigh et al., 2020) (Aus)	A medical inpatient unit at a tertiary hospital.	Medication history review & reconciliation, Patient discharge education	Advanced Pharmacy Assistants (APA) documented Medication Management Plans and Discharge Medication Records, reconciled medication histories, and managed patients own medicine bedside drawer.	8 weeks: 318 admissions and 266 discharges	Pharmacist	A minimum qualification of a Certificate IV in Hospital Health Services Pharmacy Support.	4 weeks of training and 2 weeks of lead-in
(Rathbone et al., 2020) (UK)	Six wards across 2 hospitals.	Medication preparation & administration support	Medicine Assistants (MA) were directed to support medication administration: obtaining required medicines, obtaining water for patients to take medications with, and preparing packaged medications.	4 weeks: 113 patients	Nurse	Not reported	Not reported
Authors, Year (country)	Setting	PTs task	Description PT task	Frequency PTs tasks completed	Comparator healthcare professional	Pre-service training of PTs	Additional training PT before intervention
(Seaton and Adams, 2010) (Aus)	A medical ward in a rural/regional hospital.	Medication supply & dispensing management	Implementation bedside medication drawers. Pts tasks: checking bedside drawers, determining supply requirements, dispensing and processing patients own medications, returning medication to bedside drawers, and preparing pre-packs.	2 ‘drawer’ audits in 2 different months	Nurse	A minimum qualification of a Certificate III in Health Service Assistance (and working towards Certificate IV).	Not reported
(Silverio et al., 2020) (UK)	2 specialist wards in a children’s hospital.	Medication preparation & administration support, Medication supply & dispensing management	Technician Enhanced Administration of Medications (TEAM) PT role: second checking before administration, order and re-stock medication, and readying medications for patient discharges.	3 months	Nurse	The TEAM PT held the necessary qualifications, held relevant registrations for PTs in the UK and had completed an Accredited Checking Pharmacy Technician Course.	The TEAM PT received in-house training on variety of skills by the pharmacy department within the hospital.
(Sinclair et al., 2018) (UK)	Haematology oncology ward of a university acute care children’s hospital.	Medication preparation & administration support	PTs were assigned to the busiest medicine round to support intravenous preparation and administration: second checking, preparation intravenous medication and their administration.	304 h over 76 days: 509 intravenous preparations and administrations assisted by PTs	Nurse	Not reported	30 h delivered by senior nurse trainers: intravenous management including syringe drivers and an introduction to nurse basic patient assessment skills.
(Smith and Mango, 2013) (USA)	An adult community hospital.	Medication history review & reconciliation	Dedicated medication reconciliation technicians obtained medication histories during peak admission hours.	360 medication charts obtained by PTs	Pharmacist	Not reported	Not reported
(Uhlenhopp et al., 2020) (USA)	A tertiary care hospital.	Medication history review & reconciliation	Medication History Technicians (MHTs) obtained the medication history on adult patients that manage their own medications admitted to the hospital.	817 patients	Nurse	High school diploma or GED, 3000 h of PT experience, national certification trough either the Pharmacy Technician Certification Board or National Healthcare association	Passing scores on 10 direct (witnessing the trainee obtaining the medication history) and 10 indirect observations (comparing the medication history obtained by the trainee against the one obtained by the trainer) were required before a PT could operate as an MHT. Annually shortened retesting protocol.
(Wagner et al., 2010) (USA)	Paediatric intensive care and paediatric cardiothoracic unit of a children’s hospital.	Medication supply & dispensing management	Implementation of a Medication Manager program in which pharmacy technicians delivered medications directly to locked bedside cabinets.	24 days, daily from 7 am to 12 am (24 622 medication doses)	Nurse	Not reported	Not reported

UK = United Kingdom, USA = United States of America, Aus = Australia, PT = Pharmacy Technician.

### 3.4 Tasks performed by pharmacy technicians

All 21 included studies examined the delegation of tasks—traditionally performed by other healthcare professionals—to pharmacy technicians. The most frequently reported task was medication history review and reconciliation, described in 11 studies (e.g. Buck et al., 2013; Nguyen et al., 2017; Raleigh et al., 2020). This was followed by medication preparation and administration support (5 studies) (e.g. Baqir et al., 2015; Rathbone et al., 2020); medication supply and dispensing management (4 studies) (Kjeldsen et al., 2023; Seaton and Adams, 2010; Silverio et al., 2020; Wagner et al., 2010); and patient discharge education (3 studies) (Anderson et al., 2021; Lloyd, 2020; Raleigh et al., 2020).

Some studies examined multiple domains of delegated practice and are consequently discussed under each relevant domain according to the specific tasks evaluated. This overlap highlights the multifaceted nature of pharmacy technician roles and suggests that task boundaries are often fluid rather than strictly delineated in clinical practice. A complete overview of all studies and task categories is provided in Table 2, Table 3.

**Table 3:** Effects of pharmacy technicians’ tasks – grouped by outcome.

Outcome	Pts tasks	Authors, Year	Effect
**Medication-error incidence**	*Medication history review & reconciliation*	(Chan et al., 2015) ⬤	Medication safety: No increase in clinically significant discrepancies
		(Kramer et al., 2014) ⬤	Nurses had the highest discrepancy rate per medication (0.59) compared with PTs (0.36) and pharmacists (0.16), *p* < 0.001 * Pharmacists corrected significantly more discrepancies per participant (6.39 vs 0.48 for nurses, *p* < 0.001) * PTs provided a more reliable baseline than nurses, but pharmacist verification remained essential
		(Raleigh et al., 2020) ⬤	Missed doses reduced from an average of 16.5 → 1 per round Documented interventions rose from 3 → 58 (13 by PT)
		(Buck et al., 2013) ◐	Reconciliation discrepancies: 629 detected (∼3 per patient); 45% accepted/acted on Prescribing errors: 860 detected; 96% accepted/acted on
		(Kram et al., 2019) ◐	PT involvement identified multiple discrepancies (median 3 per patient) 78% of patients had ≥1 clinically relevant discrepancy Most frequent issues: omissions and incorrect/discontinued medications; several classified as significant or serious
		(Uhlenhopp et al., 2020) ◐	PTs identified an average of 6.1 discrepancies per patient (82% omissions, 59% commissions, 50% dosing errors) PTs required 28.5 min per history
	*Medication preparation & administration support*	(Baqir et al., 2015) ⬤	Proportion of patients with ≥ 1 unacceptable omitted dose (UOD): 1,1% (intervention) vs 18,5% (control), *p* < 0.0001 * Proportion of patients with ≥ 1 critical omitted dose (COD): 1.1% (intervention) vs 7,4% (control), *p* = 0.03 * Absolute risk reduction for UOD: 17,4% (Number needed to treat = 6)
		(Rathbone et al., 2020) ⬤	Proportion of patients with > 1 unacceptable omitted dose decreased from 22% to 10%
		(Silverio et al., 2020) ◯	240 administration-related incidents/near-misses recorded over two 3-month periods Incidents would mostly not have been captured in standard hospital reporting
		(Sinclair et al., 2018) ◯	IV preparations/administrations assisted: 509 (45% of total) Near-misses prevented: 1 – 3 per day; 15 recorded in one week Patient harm: none reported
		(El-Fahimi et al., 2020) ◯	Omitted doses decreased from 14% to 5% within 3 months of implementation No medication-related harm incidents were reported
	*Medication supply & dispensing management*	(Seaton and Adams, 2010) ◐	Missed doses reduced from approximately 8–9% to 0% post-implementation (*p* < 0.01) * Improved identification of medication discrepancies
		(Kjeldsen et al., 2023) ◐	Trend toward fewer medication errors and harmful events
		(Silverio et al., 2020) ◯	81 supply-related incidents identified Incidents would mostly not have been captured in standard hospital reporting
	*Patient discharge education*	(Lloyd, 2020) ⬤	PTs had a lower overall transcription error rate than doctors (3.8%vs 18,7%) Lower frequencies across nearly all error types: dosing, omissions, writing, clinical safety Fewer minor (1,4% vs 9,4%) and significant errors (2.4%vs 9.1%), no difference in serious errors
**Pharmacy staff efficiency**	*Medication history review & reconciliation*	(Elliott et al., 2014) ⬤	Pharmacist clinical time: 58.0% → 73.9% Unpaid pharmacist overtime: 58.4 → 13.4 min/day Pharmacist interventions: 4 → 9 per day
		(Chan et al., 2015) ⬤	Reconciliation completeness: 61% → 91% after PT involvement History completed ≤48 h: 82% → 100% (p = 0.030) *
		(Hwang et al., 2021) ⬤	Medication reconciliation completion increased 21% → 89% with PT involvement Reconciliation within 24 h improved 7% → 75%
		(Jobin et al., 2018) ⬤	High mean accuracy of PT histories (92.9%) 69% of PT-obtained histories were error-free No effect of age, medication count, or shift on accuracy (all *p* > 0.05)
		(Nguyen et al., 2017) ◐	Mean time per patient: pharmacists 58.5 min vs PTs 79.4 min (*p* = 0.14) PTs required an additional 26 min of pharmacist supervision
		(Smith and Mango, 2013) ◐	Accuracy increased from 45.8% to 95% (*p* < 0.001) * Reconciliation completeness increased from 44.2% to 92.8% (*p* < 0.001) * Missing medications decreased from 9.2% to 0.8% PTs required 33 min per history, pharmacists required 5 min for review
	*Patient discharge education*	(Raleigh et al., 2020) ⬤	Medication management plans documented increased from 43.3% → 52.8% (p = 0.019) * Discharge medication records documented increased from 63.0% → 78.6% (p < 0.001) * Clinical pharmacy reviews increased by 35%
**Nursing efficiency**	*Medication history review & reconciliation*	(Buck et al., 2013) ◐	Nursing time: 33–38% reduction in medication administration time
	*Medication preparation & administration support*	(Rathbone et al., 2020) ⬤	Duration of morning medication rounds reduced from 74,5 min to 60,8 min (p = 0002) * Average of 17,4 nursing hours released per ward per week
		(El-Fahimi et al., 2020) ◯	Nursing efficiency reportedly improved: fewer interruptions, smoother medication rounds
	*Medication supply & dispensing management*	(Seaton and Adams, 2010) ◐	Nursing staff reported higher efficiency and perceived improvements in medication safety
		(Wagner et al., 2010) ◐	Nursing trips to medication room decreased by 85%, time spent reduced by 45% Improved communication and coordination between nursing teams and pharmacy
		(Kjeldsen et al., 2023) ◐	PTs reduced nurses’ dispensing time by approximately 1.4h/day Fewer interruptions during medication dispensing (3 – 2,6 per day vs 19,4)
**Staff satisfaction**	*Medication history review & reconciliation*	(Elliott et al., 2014) ⬤	Improved pharmacists’ satisfaction
		(Raleigh et al., 2020) ⬤	Higher satisfaction reported by both pharmacists and assistants
	*Medication preparation & administration support*	(Sinclair et al., 2018) ◯	Nursing workload reduced, freed time for direct patient care
	*Medication supply & dispensing management*	(Kjeldsen et al., 2023) ◐	Nursing staff reported higher satisfaction and perceived improvements in patient safety
	*Patient discharge education*	(Raleigh et al., 2020)⬤	Higher satisfaction reported by both pharmacists and assistants
**Cost-effectiveness**	*Medication history review & reconciliation*	(Nguyen et al., 2017) ◐	Cost per patient: pharmacists $55.91 vs PTs $45.00 (*p* = 0.32) No statistically significant difference in cost, but PTs were overall less expensive
	*Medication supply & dispensing management*	(Seaton and Adams, 2010) ◐	Cost-savings of 5–11% per quarter trough reduced wastage and reuse of returned medicines
		(Wagner et al., 2010)	Missing medications reduced by 85% resulting in approximately $42,000 annual savings
**Patient satisfaction**	*Patient discharge education*	(Anderson et al., 2021) ◐	Higher ‘very satisfied’ scores for information and ease (92–97% vs 79–86%) with discharge education by PT. Overall satisfaction slightly higher (94%vs 86%), confidence in medication use similar (95%) More written info, longer education (7 vs 3 min), most questions answered independently

PT = Pharmacy Technician ⬤ high quality; ◐ moderate quality; ◯ low quality.

#### 3.4.1 Medication history review and reconciliation

Eleven studies evaluated pharmacy technicians’ involvement in medication history collection and reconciliation across diverse hospital settings (e.g. Elliott et al., 2014; Jobin et al., 2018; Smith and Mango, 2013). Collectively, these studies demonstrated that pharmacy technician participation improved the accuracy, completeness, and timeliness of medication histories and reconciliations, while contributing to reductions in medication discrepancies and enhanced workflow efficiency across disciplines. Reported outcomes fell under the domains of medication error incidence, staff satisfaction, cost-effectiveness, and pharmacy and nursing efficiency.

In several studies, pharmacy technician-led or pharmacy technician-supported processes substantially reduced discrepancies compared with standard nurse- or physician-led practices. Buck et al. (2013) reported 629 reconciliation discrepancies (∼3 per patient) and 860 prescribing errors, of which 45% and 96%, respectively, were accepted and corrected by prescribers. Their involvement was also associated with a 33–38% reduction in nurses’ medication administration time. Kramer et al. (2014) found significantly fewer inaccuracies for pharmacy technicians compared with nurses (0.36 vs 0.59 discrepancies per medication, *p* < 0.001), while pharmacists achieved the highest accuracy (0.16 discrepancies per medication). In intensive care and geriatric populations, Kram et al. (2019) and Uhlenhopp et al. (2020) demonstrated that pharmacy technicians identified multiple clinically relevant discrepancies per patient, most frequently omissions and dosing errors. These findings support the feasibility and clinical value of technician-led medication history collection even in complex care settings.

Significant improvements in timeliness and process efficiency were also observed. In a rural Australian hospital, Hwang et al. (2021) reported that implementation of ward-based pharmacy technicians increased medication reconciliation completion from 21% to 89%, and reconciliation within 24 h of admission improved from 7% to 75%. Similarly, Smith and Mango (2013) demonstrated that a pharmacy-based reconciliation model - in which pharmacy technicians collected pre-admission medication histories and pharmacists verified and reconciled the information at hospital administration - increased documentation accuracy from 45.8% to 95% and reconciliation completeness from 44.2% to 92.8%, while missing medications decreased from 9.2% to 0.8%. The average time per history was 33 min for technicians and 5 min for pharmacists.

Economic and staffing outcomes were also favourable. In a cost analysis, Nguyen et al. (2017) reported that pharmacist-supervised pharmacy technicians achieved comparable efficiency to pharmacists (79.4 vs 58.5 min per patient, *p* = 0.07) at a lower total labour cost ($45.00 vs $55.91 per patient). Elliott et al. (2014) found that introducing an expanded ward pharmacy technician role increased pharmacists’ clinical task time from 58.0% to 73.9% (*p* < 0.0001) and reduced unpaid overtime from 58.4 to 13.4 min per day (*p* < 0.0001). The number of pharmacist interventions to resolve medication-related problems more than doubled (from four to nine per day, *p* < 0.0001), accompanied by improvements in staff satisfaction and no increase in reported medication incidents. Raleigh et al. (2020) similarly reported an increase in completion of medication histories and reconciliations from 43.3% to 52.8% (*p* = 0.019), alongside higher pharmacist and technician satisfaction and fewer missed medication doses (16.5 to 1 per round).

Accuracy outcomes were consistently high. Jobin et al. (2018) found a mean accuracy of 92.9% (±14.2%) in pharmacy technician-obtained medication histories, with no significant effect of patient age, number of medications, or admitting shift (all p > 0.05). In paediatric settings, Chan et al. (2015) reported that reconciliation completeness during intrahospital transfers increased from 61% to 91%, with medication history completion within 48 h improving from 82% to 100% (p = 0.030) and no increase in clinically significant discrepancies.

All of these eleven studies - who evaluated pharmacy technicians’ involvement in medication history collection and reconciliation - are rated as moderate to high quality.

#### 3.4.2 Medication preparation and administration support

Five studies evaluated pharmacy technicians’ involvement in direct support of medication preparation and administration support on hospital wards. In these studies, pharmacy technicians were embedded within nursing medication rounds to assist with medicine preparation and dose checking (e.g. Baqir et al., 2015; El-Fahimi et al., 2020).

Rathbone et al. (2020) implemented ward-based pharmacy technicians on six hospital wards in the United Kingdom. Technicians supported nurses during scheduled medication rounds by sourcing medicines and prompting administration documentation. This intervention was associated with a significant reduction in the duration of morning medication rounds (mean 74,5 to 60,8 min, p = 0002), and the proportion of patients with more than one unacceptable omitted dose decreased from 22% to 10%, alongside an average release of 17.4 nursing hours per ward per week.

Baqir et al. (2015) trained pharmacy technicians to work alongside nurses during medication administration on acute medical and surgical wards. The proportion of patients experiencing at least one unacceptable omitted dose was significant lower in the intervention group compared with control wards (1.1%vs 18.5%, p < 0,0001). Critical omitted doses were also reduced (1.1%vs 7.4%, p = 0.03).

El-Fahimi et al. (2020) introduced ward-based pharmacy technicians into an acute admission unit, where they administered oral and intravenous medicines and maintained controlled drug records. Omitted doses decreased from 14% to 5% within three months of implementation and no medication-related harm incidents were reported during the study period. In addition, although not statistically evaluated, El-Fahimi et al. (2020) reported perceived improvements in nursing efficiency, with fewer interruptions and smoother completion of medication rounds.

Silverio et al. (2020)described a ward-based pharmacy technician role in a children’s hospital. The technician took part in second-checking medicines prior to administration, supported nursing staff by clarifying dosing, side effects, and administration requirements. They maintained a personal incident log, which recorded 240 administration-related incidents/near-misses, many of which would not otherwise have been captured in the hospitals’ reporting systems.

Similarly, on a busy paediatric oncology ward in the United Kingdom, Sinclair et al. (2018) trained pharmacy technicians to assist with the preparation and administration of intravenous medications, acting as second checkers alongside nurses. This intervention prevented 1 – 3 near-miss incidents per day and nursing staff reported that the integrated pharmacy technician role reduced workload and task pressure during medication rounds and freed additional time for direct patient care.

Rathbone et al. and Baqir et al. were higher-quality studies demonstrating statistically significant improvements, while the remaining three studies were lower-quality studies.

#### 3.4.3 Medication supply and dispensing management

Four studies examined pharmacy technicians’ involvement in medication supply and dispensing management on hospital wards (e.g. Kjeldsen et al., 2023; Wagner et al., 2010).

In a Danish pilot study from Kjeldsen et al. (2023), pharmacy technicians were responsible for dispensing and preparing medications on a geriatric ward, ensuring availability and supporting nursing staff in their medication tasks. Their involvement reduced nurses’ dispensing time by approximately 1.4 h per day, fewer interruptions during the process, and showed a tendency toward fewer medication errors. Nursing staff reported higher satisfaction and perceived improvements in patient safety as a result of the service.

In Australia, Seaton and Adams, 2010 evaluated a ward-based pharmacy technician-facilitated medication delivery system on a 30-bed medical ward. Technicians managed the ward’s medication stock, checked bedside medication drawers, ensured timely supply of additional (non-stock) medicines, and removed stopped or unused medications. After implementation, the proportion of regularly prescribed doses that were missed decreased from approximately 8–9% before implementation to 0% afterwards (p < 0,01). The intervention also improved the identification of medication discrepancies and generated cost savings of 5–11% of the ward’s quarterly medication budget, mainly through reduced wastage and the reuse of returned medicines. Nursing staff reported greater efficiency and perceived improvements in medication safety.

In the United States, Wagner et al. (2010) implemented a “Medication Manager” program in two paediatric intensive care units. Pharmacy technicians retrieved medications from the pharmacy and delivered them directly to secure bedside cabinets. The aim was to reduce nurses’ medication collection time and to minimise missing doses. After implementation, nursing trips to the medication room decreased by 85%, and the time spent there was reduced by 45%. The number of missing medications and the associated costs fell by approximately 85%. Nursing staff reported greater satisfaction and more time available for direct patient care, while pharmacists noted improved communication and coordination between pharmacy and nursing teams.

Silverio et al. (2020), previously discussed under medication preparation and administration support, also reported that the pharmacy technician undertook ward-based medication supply management. The pharmacy technician identified and followed up supply-related issues, including missing or unavailable medicines and anticipated shortages. A total of 81 supply-related incidents were detected. No changes in medication availability were assessed; the study described detection and documentation of supply-related issues only.

All the studies in this domain were of moderate to low methodological quality.

#### 3.4.4 Patient discharge education

Three studies examined the involvement of pharmacy technicians in patient discharge processes, with a focus on medication education and the preparation of discharge documentation (Anderson et al., 2021; Lloyd, 2020; Raleigh et al., 2020). Raleigh et al. (2020), previously discussed under medication history review and reconciliation, also evaluated pharmacy technicians’ involvement in discharge processes. Pharmacy technicians prepared discharge medication records (DMRs) under pharmacist supervision, resulting in an increase in documented DMRs from 63,0% to 78.6% (p < 0001) and greater pharmacist efficiency and satisfaction.

Lloyd (2020) examined the accuracy of transcribing discharge medication prescriptions and found that pharmacy technicians had a lower transcription error rate than physicians (3.8%vs 18.7%, p < 0.05). The most frequent error types were dosing errors, omissions, and clinical safety-related discrepancies, with lower frequencies observed in the technician group across nearly all categories.

Anderson et al. (2021) compared discharge medication education provided by a pharmacy technician with that provided by a pharmacist in a short-stay surgical setting. Patient satisfaction was high in both groups; however, a higher proportion of patients counselled by the technician reported being ‘very satisfied’ with the amount of information provided (92%vs 79%) and with the ease of following the education (97%vs 86%).

The studies in this domain were assessed as moderate to high quality.

### 3.5 Patient-, pharmaceutical-, nursing-, and ward-related outcomes measured for pharmacy technician tasks

The included studies reported a range of outcomes associated with tasks performed by pharmacy technicians, encompassing effects on patients, pharmaceutical processes, and ward-level functioning. Reported outcomes included medication‑error incidence (13 studies) (e.g. Buck et al., 2013; Rathbone et al., 2020), pharmacy staff efficiency (8 studies) (e.g. Chan et al., 2015, ; Elliott et al., 2014), nursing efficiency (6 studies) (e.g. El-Fahimi et al., 2020; Kjeldsen et al., 2023), staff satisfaction (5 studies) (e.g. Kjeldsen et al., 2023; Sinclair et al., 2018), cost‑effectiveness (3 studies) (Nguyen et al., 2017; Seaton and Adams, 2010; Wagner et al., 2010), and patient satisfaction (1 study) (Anderson et al., 2021), with several studies reporting on multiple outcomes (Table 3, Table 4).

**Table 4:** Relationship between pharmacy technician tasks and measured outcomes.

PT Tasks →	Medication history review & reconciliation	Medication preparation & administration support	Medication supply & dispensing management	Patient discharge education
Measured outcomes ↓				
**Medication-error incidence**	**+** Chan et al. **∼** Kram et al. **+** Kramer et al. * **+** Uhlenhopp et al. **+** Buck et al. **+** Raleigh et al.	**+** Baqir et al. * + El-Fahimi et al. **+** Rathbone et al. **∼** Silverio et al. **+** Sinclair et al.	**+** Seaton et al. * ∼ Silverio et al. **+** Kjeldsen et al.	**+** Lloyd et al. *
**Pharmacy staff efficiency**	**+** Elliot et al. * + Chan et al. * + Hwang et al. **∼** Jobin et al. **∼** Nguyen et al. * + Raleigh et al. * + Smith et al. *			**+** Raleigh et al. *
**Nursing efficiency**	**+** Buck et al.	**+** El-Fahimi et al. **+** Rathbone et al. *	**+** Seaton et al. **+** Wagner et al. **+** Kjeldsen et al.	
**Staff Satisfaction**	**+** Elliot et al. **+** Raleigh et al.	**+** Sinclair et al.	**+** Kjeldsen et al. **+** Wagner et al.	**+** Raleigh et al.
**Cost-effectiveness**	**∼** Nguyen et al. *		**+** Seaton et al. **+** Wagner et al.	
**Patient satisfaction**				**+** Anderson et al.

+ positive effect∼ no difference * statistically significant PT = Pharmacy Technician.

Pharmacy technician tasks were associated with measurable improvements across multiple outcome domains (Table 3, Table 4). *Medication-error incidence* was reduced through medication history review and reconciliation, with pharmacy technicians identifying omissions, dosing errors, and prescribing discrepancies, many of which were acted upon. Support in medication preparation, administration, and supply management further decreased omitted or delayed doses, prevented near-misses, and improved overall medication safety. Pharmacy technician-led discharge education also enhanced transcription accuracy and reduced minor and significant errors in medication records (Lloyd, 2020).

*Pharmacy staff efficiency* improved through more complete and accurate medication histories, optimised pharmacist time, and increased documentation of clinical interventions. *Nursing efficiency* benefitted from reduced administration time, shorter medication rounds, fewer interruptions, and decreased time spent on dispensing tasks.

*Staff satisfaction* was consistently higher among both pharmacy and nursing staff when pharmacy technicians were involved in medication histories, administration support, supply management, and discharge education. *Cost-effectiveness* analyses suggested that pharmacy technician involvement was either cost-neutral or led to savings through reduced missed doses and lower wastage. *Patient satisfaction* was positively influenced by discharge education, with patients reporting improved understanding of medications and higher overall satisfaction.

## 4 Discussion

This is the first systematic review that comprehensively synthesised evidence on the tasks performed by pharmacy technicians and their effects on patient-, pharmaceutical-, nursing-, and ward-related outcomes in hospital settings. Across the 21 included studies, pharmacy technician involvement consistently improved medication history accuracy and completeness (e.g. Buck et al., 2013; Chan et al., 2015; Elliott et al., 2014), reduced discrepancies and errors (e.g. Baqir et al., 2015; Kramer et al., 2014), supported medication preparation and administration (e.g. El-Fahimi et al., 2020; Rathbone et al., 2020), optimised medication supply and dispensing management (e.g. Kjeldsen et al., 2023; Seaton and Adams, 2010), and enhanced discharge processes (Anderson et al., 2021; Lloyd, 2020; Raleigh et al., 2020). In addition to improving patient-related outcomes, pharmacy technician involvement positively affected healthcare teams and processes, including reducing nursing workload and interruptions during medication rounds (e.g. Kjeldsen et al., 2023; Rathbone et al., 2020), increasing pharmacists’ capacity to focus on clinical interventions, and enhancing overall workflow efficiency (e.g. Hwang et al., 2021; Jobin et al., 2018). Staff satisfaction was reported to increase (e.g. Sinclair et al., 2018; Wagner et al., 2010), and several studies suggested cost-effectiveness, with reductions in medication wastage and missed doses, without compromising care quality (Nguyen et al., 2017; Seaton and Adams, 2010; Wagner et al., 2010). However, given the substantial heterogeneity and generally moderate to low methodological quality of the included studies, these results should be interpreted with caution. Collectively, these findings indicate that pharmacy technicians can safely and effectively extend the capacity of ward-based care when supported by appropriate training, role clarity, and supervision.

Building on prior research demonstrating the effectiveness of pharmacy technicians in advanced pharmacy roles - particularly in centralised medication preparation, dispensing, and reconciliation - this review positions ward-based pharmacy technician contributions within a broader international trend. Previous systematic reviews report that many healthcare systems, including in the UK, USA, Canada and Australia, increasingly advocate for task delegation to pharmacy technicians to optimise workflow, relieve pressure on pharmacists and nurses, and improve patient safety. Mattingly and Mattingly (2018) highlighted that international expansion of advanced pharmacy technician roles has been supported by structured training, certification programmes, and role redesign initiatives that allow pharmacy technicians to take on increasingly technical, administrative, and patient-facing responsibilities. Similarly, Banks et al. (2020) show that advanced-scope pharmacy technician roles provide substantial institutional benefits, including significant time savings for pharmacists and wage-related cost reductions when tasks such as medication history taking, accuracy checks, and ward-based supply functions are delegated appropriately. The present review extends these insights by demonstrating that similar benefits occur when pharmacy technicians are integrated directly into ward-based medication management and patient care pathways, although the current evidence base remains limited and heterogeneous.

Interpretation of this evidence requires consideration of the substantial heterogeneity in pharmacy technician role definitions, training, and supervision reported across studies. Differences in job titles—such as pharmacy assistant, ward-based technician, pharmaconomist, or advanced pharmacy technician—reflect national differences in workforce structures and scopes of practice. Prior literature indicates that this variability is further shaped by wide differences in educational pathways and competency requirements. Mattingly and Mattingly (2018) identified six distinct training mechanisms used internationally, including certification, prior work experience, formal training programmes, informal on-the-job learning, and test-based competency assessments; most advanced roles required a combination of these elements, illustrating the heterogeneity in role preparation. They further noted that interstate and intercountry differences in legal entry requirements and authorised activities may influence both the tasks pharmacy technicians can perform and the extent to which pharmacists feel comfortable delegating responsibilities.

Many studies in this review did not report the educational background, or competency requirements of participating pharmacy technicians, limiting interpretability and preventing determination of whether differences in outcomes were attributable to differences in training, oversight, or to task delegation itself. Similarly, Banks et al. (2020) reported that 11 of the 16 included studies delivered structured preparatory training for advanced-scope roles—such as self-paced learning, supervised practical training and competency assessments—highlighting that safe delegation depends on clear educational frameworks. Collectively, these findings indicate that although pharmacy technicians can meaningfully contribute to ward-based care, the variability in required training and competencies complicates direct comparison of outcomes across studies. Standardisation of reporting on pharmacy technician qualifications, training, and supervision would therefore substantially strengthen future evidence.

Taken together, these contextual and role-related factors shape how the available evidence should be interpreted. However, the findings across studies point consistently in the direction of benefit, with pharmacy technician involvement commonly associated with fewer medication-related errors, more efficient medication workflows and improved staff experiences. Given the predominance of observational evidence, these should be interpreted as suggestive and directionally consistent indications of benefit, rather than definitive causal effects, as residual confounding, selection bias, and variability in implementation contexts may have influenced the observed associations. Moreover, the absence of controlled comparisons in several studies limits the ability to attribute observed improvements solely to pharmacy technician involvement.

Beyond methodological considerations, a further consideration relates to how the redistribution of medication-related tasks intersects with traditional domains of nursing practice. Several of the activities performed by pharmacy technicians in the included studies—such as medication preparation, second checking, reconciliation- and discharge-related communication—overlap with core nursing responsibilities for ensuring safe medication administration. While the reviewed studies generally reported improved workflow efficiency and fewer administration errors, the delegation of such tasks also raises questions about professional accountability, interprofessional communication and the potential risk of care fragmentation, particularly in complex care pathways where multiple professionals contribute to shared medication processes. These dynamics should be understood within the broader framework of healthcare systems as complex adaptive systems, in which task redistribution may have intended and unintended effects on care processes.

To maintain patient safety, any expansion of pharmacy technician roles should therefore be embedded within clear delegation protocols, structured supervision arrangements and well-defined boundaries for tasks requiring nursing clinical judgement. Joint training for nurses and pharmacy technicians, as well as shared decision-making during medication rounds, may help maintain coordination and avoid overreliance on technicians for tasks that necessitate nursing assessment skills. These safeguards are essential to ensure that task redistribution enhances, rather than compromises, medication safety and continuity of care within complex multidisciplinary environments and should be aligned with established patient safety frameworks.

Taken together, these considerations inform broader implications for practice. From a practical perspective, these findings underline the value of integrating pharmacy technicians into ward-based medication management. Their involvement can improve patient safety through more accurate medication histories and reduced discrepancies, while simultaneously relieving workload pressures on nursing and pharmacy staff. By delegating technical and distributive tasks to pharmacy technicians, nurses can prioritise direct patient care and pharmacists can focus on clinical decision-making and medication optimisation. To ensure safe and effective task delegation, clear role delineation, competency-based training, and structured supervision remain essential. Consistent reporting of pharmacy technician training, responsibilities, and oversight would further support benchmarking and guide broader implementation across hospitals. These implications highlight the potential for healthcare organisations and policymakers to strategically expand ward-based pharmacy technician roles to strengthen workflow efficiency and optimise the use of clinical pharmacy resources.

Future studies should employ stronger methodological designs - including controlled comparisons, standardised outcome measures, and transparent reporting - to strengthen the reliability and comparability of the evidence. In addition to patient-related outcomes, studies should focus on clearly demonstrating the impact of pharmacy technicians on nursing workflow in medication management tasks performed directly on the hospital ward, including time savings, reduction in medication preparation errors, and smoother medication rounds. By quantifying these operational benefits, studies can provide robust evidence of pharmacy technicians as a supportive workforce for nursing staff, clarifying the extent to which ward-based task delegation enhances efficiency and patient safety. Evaluating interprofessional communication and coordination will further inform strategies to optimise collaboration and ensure safe, effective pharmacy technician task allocation on hospital wards. Future implementation research should also consider workforce capacity, as shortages of trained pharmacy technicians in some settings may influence the feasibility of expanding ward-based roles.

## 5 Limitations

This review has methodological strengths, including a comprehensive multi-database search, screening by multiple authors, and structured quality assessment. Limitations include variability in study quality, small sample sizes, single-centre settings, and heterogeneity in study designs and outcome measures, which hindered meta-analysis and limited the ability to directly compare studies. Additionally, the predominance of observational designs restricts causal inference regarding the true effects of pharmacy technician integration on hospital wards. Despite these limitations, the review provides meaningful insights into pharmacy technician contributions and identifies areas for targeted future research.

In addition, because of this heterogeneity in design, outcomes and intervention characteristics, a formal certainty of evidence assessment (e.g., GRADE based approaches for observational studies) were not feasible. Interpretation therefore relied on a structured narrative comparison of methodological strengths and weaknesses across studies. Across the major outcome domains (medication error outcomes, pharmacy efficiency, nursing efficiency, satisfaction and cost related outcomes), the overall certainty of the evidence should be considered moderate to low, and findings should be interpreted with appropriate caution.

The methodological quality of the included studies also influenced the strength of conclusions across outcome domains. For medication error outcomes, findings were generally consistent but often limited by incomplete adjustment for confounding. Evidence for nursing and pharmacy efficiency showed similar directional patterns across studies but was frequently based on single-centre observational designs, reducing confidence in effect magnitude. Satisfaction outcomes relied on small samples with limited methodological detail, whereas cost related outcomes were derived from heterogeneous economic methods. As such, results—while directionally consistent—should be interpreted with caution, particularly in domains where lower quality studies predominated.

## 6 Conclusion

Across a wide range of international studies, this review found suggestive and directionally consistent evidence that ward-based pharmacy technicians can improve medication accuracy, enhance workflow efficiency and contribute positively to staff and patient experiences. These signals of benefit were observed across multiple outcome domains. However, the strength of inference is limited by the predominance of observational designs, methodological heterogeneity and inconsistent outcome definitions, which constrain the ability to draw firm conclusions regarding effect size or causality.

Future research should therefore prioritise controlled, multicentre study designs with standardised outcome measures to confirm the magnitude, reproducibility and cost-effectiveness of these effects across different healthcare systems. Such evidence is essential to inform safe implementation, professional role delineation and workforce planning for pharmacy technicians on hospital wards and to support evidence-based policy development at institutional and national levels.

## Funding

This research did not receive any specific grant from funding agencies in the public, commercial, or not-for-profit sectors.

## Declaration of generative AI and AI-assisted technologies in the manuscript preparation process

During the preparation of this work, the authors used Microsoft Copilot to assist with language and readability improvements for short passages. After using this tool, the authors reviewed and edited the content as needed and take full responsibility for the content of the published article.

## CRediT authorship contribution statement

**Marjan De Graef:** Writing – original draft, Visualization, Resources, Project administration, Methodology, Investigation, Formal analysis, Data curation, Conceptualization. **Jolien Heirman:** Visualization, Methodology, Investigation, Formal analysis, Data curation. **Tamara Snoeck:** Investigation, Data curation. **Eibert R. Heerdink:** Writing – review & editing, Validation, Supervision, Methodology, Conceptualization. **Nienke E. Dijkstra:** Writing – review & editing, Visualization, Validation, Supervision, Methodology, Data curation, Conceptualization. **Tinne Dilles:** Writing – review & editing, Visualization, Validation, Supervision, Resources, Methodology, Data curation, Conceptualization. **Brecht Serraes:** Writing – review & editing, Validation, Supervision, Methodology, Investigation, Conceptualization.

## Declaration of competing interest

The authors declare that they have no known competing financial interests or personal relationships that could have appeared to influence the work reported in this paper.

## Data Availability

No new data were generated or analysed in this study. This systematic review is based solely on previously published research. As no original datasets were created, research data cannot be shared.

## Appendix Supplementary materials

Image, application 1

Image, application 2

Image, application 3
