# Supplementary material for: Roles and impact of pharmacy technicians on hospital wards: a systematic review
Source: Int J Nurs Stud Adv. 2026 Jun 10;11:100593. doi: 10.1016/j.ijnsa.2026.100593 (PMC13292472; doi:10.1016/j.ijnsa.2026.100593)
Supplement: Supplementary file 3 [file mmc3.docx]

**Supplementary Table S2.**

The detailed search strategies used for each database are presented below. Search terms were combined using Boolean operators (AND, OR). Truncation (*) and phrase searching (“ ”) were applied where appropriate. The strategy was adapted to each database’s syntax and controlled vocabulary (e.g., MeSH in PubMed, Emtree in Embase, CINAHL Headings in CINAHL).

| **Database** | **Platform** | **Last search date** | **Search String** | **Filters/limits** |
| --- | --- | --- | --- | --- |
| **PubMed - Medline** | NCBI | 30 Nov 2024 | (“Pharmacy Technicians”[MeSH Terms] OR “pharmacy assistant”[ Title/Abstract] OR “pharmacy assistants”[ Title/Abstract] OR “Pharmacy Technicians”[ Title/Abstract] OR “pharmacy technician”[ Title/Abstract] OR “pharmacy support”[ Title/Abstract] OR “dispensary technician”[ Title/Abstract]) AND (“Hospitals”[MeSH Terms] OR “nursing ward”[Title/Abstract] OR “hospital unit”[Title/Abstract] OR “hospital ward”[Title/Abstract] OR “Hospitals”[Title/Abstract] OR “Hospital”[Title/Abstract]) | Language: EN/FR/NL; full-text; No date limits |
| **Embase** | Elsevier | 30 Nov 2024 | (‘pharmacy technician’/exp OR ‘pharmacy assistant’/exp OR ‘pharmacy support’:ab,ti OR ‘dispensary technician’:ab,ti) AND (‘hospital’/exp OR ‘hospital unit’:ab,ti OR ‘hospital ward’:ab,ti OR ‘nursing ward’:ab,ti) | Language: EN/FR/NL; full-text; No date limits |
| **CINAHL** | EBSCOhost | 30 Nov 2024 | (“pharmacy technician” OR “pharmacy assistant” OR “pharmacy assistants” OR “pharmacy support” OR “dispensary technician”) AND (“hospital” OR “hospital ward” OR “nursing ward” OR “hospital unit”) | Language: EN/FR/NL; full-text; No date limits |
| **Web of Science** | Clarivate | 30 Nov 2024 | TS=(“pharmacy technician” OR “pharmacy assistant” OR “pharmacy support” OR “dispensary technician”) AND TS=(“hospital” OR “hospital ward” OR “nursing ward” OR “hospital unit”) | Language: EN/FR/NL; No date limits |
| **ScienceDirect** | Elsevier | 30 Nov 2024 | TITLE-ABSTR-KEY (“pharmacy technician” OR “pharmacy assistant” OR “pharmacy support” OR “dispensary technician”) AND TITLE-ABSTR-KEY (“hospital” OR “hospital ward” OR “nursing ward” OR “hospital unit”) | Language: EN/FR/NL; No date limits |

Additional information sources:

- Reference lists of included studies and relevant reviews were manually screened
- Conference records retrieved through database searches were assessed where available.
- No trial registries, grey literature databases, or professional organisation websites were systematically searched.
